# ISM1 suppresses LPS-induced acute lung injury and post-injury lung fibrosis in mice

**DOI:** 10.1186/s10020-022-00500-w

**Published:** 2022-06-25

**Authors:** Ngan Nguyen, Simin Xu, Terence Yin Weng Lam, Wupeng Liao, W. S. Fred Wong, Ruowen Ge

**Affiliations:** 1 Department of Biological Sciences, Faculty of Science, National University of Singapore, Singapore, 117543 Republic of Singapore; 2 Department of Pharmacology, Yong Loo Lin School of Medicine, National University of Singapore, Singapore, 117600 Republic of Singapore; 3 Singapore-HUJ Alliance for Research and Enterprise, National University of Singapore, Singapore, 138602 Republic of Singapore; 4 Drug Discovery and Optimization Platform, National University Health System, Singapore, 117600 Republic of Singapore

**Keywords:** ISM1, LPS, Inflammation, Acute lung injury, Pulmonary fibrosis

## Abstract

**Background:**

Acute lung injury/acute respiratory distress syndrome (ALI/ARDS) are clinical syndromes characterized by acute lung inflammation, pulmonary edema and hypoxemia, with up to 50% mortality rate without effective pharmacological therapy. Following the acute inflammation, repair and remodeling occurs which in some cases resulting in lung fibrosis. The pathophysiology of ALI/ARDS remains incompletely understood. Lipopolysaccharide (LPS)-induced ALI in mice have been widely used as a model to study human ALI/ARDS. Isthmin 1 (ISM1) is a secreted protein highly abundant in mouse lung. We have previously reported that upon intratracheal LPS instillation, ISM1 expression in the lung is further upregulated. Recently, we also reported that ISM1 is an anti-inflammatory protein in the lung with *Ism1*^-/-^ mice presenting spontaneous chronic low-grade lung inflammation and obvious emphysema at young adult stage. However, what role ISM1 plays in ALI/ARDS and lung fibrosis remain unclear.

**Methods:**

Using *Ism1*^-/-^ mice and intratracheal LPS-induced ALI, and local delivery of recombinant ISM1 (rISM1), we investigated the role ISM1 plays in ALI and post-ALI lung fibrosis using flow cytometry, Western blot, antibody array, immunohistochemistry (IHC), immunofluorescent and other histological staining.

**Results:**

We reveal that ISM1 deficiency in mice led to an intensified acute lung inflammation upon intratracheal LPS challenge, with a heightened leukocyte infiltration including neutrophils and monocyte-derived alveolar macrophages, as well as upregulation of multiple pro-inflammatory cytokines/chemokines including tumor necrosis factor α (TNF-α). Although innate immune cells largely subsided to the baseline by day 7 post-LPS challenge in both wild-type and *Ism1*^*−/−*^ mice, *Ism1*^*−/−*^ lung showed increased post-ALI fibrosis from day 9 post-LPS treatment with increased myofibroblasts, excessive collagen accumulation and TGF-β upregulation. The heightened lung fibrosis remained on day 28 post-LPS. Moreover, intranasal delivered recombinant ISM1 (rISM1) effectively suppressed LPS-induced acute lung inflammation and ALI, and rISM1 suppressed LPS-induced NF-κB activation in cultured mouse alveolar macrophages.

**Conclusion:**

Together with our previous report, this work further established ISM1 as an endogenous anti-inflammation protein in the lung, restraining excessive host inflammatory response to LPS-triggered ALI and suppressing post-ALI lung fibrosis likely through suppressing NF-κB activation and pro-inflammatory cytokine/chemokine production.

**Supplementary Information:**

The online version contains supplementary material available at 10.1186/s10020-022-00500-w.

## Introduction

Upon infectious or non-infectious respiratory insults, the mammalian host mount an acute inflammatory response in the lung for self-protection. The mammalian host also has means to limit this pulmonary inflammation, eventually resolving the inflammation to prevent collateral and systemic damages. Acute lung injury (ALI) and its severe manifestation, acute respiratory distress syndrome (ARDS), are spectrum of clinical syndromes with up to 50% mortality rate without effective pharmacological therapies (Dushianthan et al. 2011; Matthay et al. 2019). ALI/ARDS is characterized by lung edema, massive inflammation, and alveolar barrier damage, leading to lung dysfunction. These symptoms are caused by increased vascular permeability, inflammatory cell infiltration (mostly neutrophils), and release of proinflammatory mediators by the infiltrating leukocytes as well as lung parenchymal cells (Wheeler and Bernard 2007). Following the acute inflammation and injury, the lung goes through repair and remodeling to regain homeostasis, with concomitant fibrosis and scar formation which either get resolved eventually or persist into a long-term lung fibrosis damage. Despite the many advances in the pathophysiology of ALI/ARDS, how lung repair and remodel to regain homeostasis and function remain poorly understood (Gill et al. 2017).

Lipopolysaccharide (LPS) is a glycolipid component of gram-negative bacteria cell wall that can evoke severe inflammatory effects in mice and human. LPS-induced ALI in experimental animals simulates acute respiratory distress syndrome in humans. Short-term intratracheal LPS challenge in mice often stimulates mixed inflammatory reaction in both the airway and lung. This includes disruption of the lung epithelial and endothelial barriers, increase in inflammatory cell infiltration and release of proinflammatory and cytotoxic mediators (Kabir et al. 2002; Matute-Bello et al. 2008; Copeland et al. 2005). These phenotypes are clinically relevant for both ALI and ARDS. Multiple intracellular signaling events are initiated upon LPS challenge. Mostly LPS binds to and signals through toll-like receptor 4 (TLR-4) complex to activate nuclear factor kappa B (NF-κB) (Kawasaki and Kawai 2014; Poltorak et al. 1998; Pålsson-McDermott and O'Neill 2004). Activated NF-κB translocate into the nucleus and stimulates the transcription of many proinflammatory cytokines including interleukin-1 (IL-1) and tumor necrosis factor-α (TNF-α) by directly binding to the consensus target sequences in their enhancer/promoter regions (Lawrence 2009; Mizgerd et al. 2002). Importantly, NF-κB is active in alveolar macrophages of ARDS patients (Schwartz et al. 1996), implicating the involvement in NF-κB signaling in the development and progression of ALI and ARDS.

Isthmin 1 (ISM1) is a secreted protein expressed at its highest level in the mouse lung, many folds higher than its expression in other organs (Zhang et al. 2011; Lam et al. 2022). It was previously identified by us as a secreted antiangiogenic and proapoptotic protein, inducing apoptosis in vascular endothelial cells through two cell surface receptors, αvβ5 integrin and cell surface glucose regulated protein of 78 kDa (csGRP78) (Zhang et al. 2011; Xiang et al. 2011; Chen et al. 2014). Subsequently, ISM1 was found to induce vascular permeability both in *vitro* and in mouse lung (Venugopal et al. 2015). Recently, we further discovered that ISM1 deficiency in mice (Ism1^−/−^) led to spontaneous low-grade lung inflammation and emphysema at young adult stage (2 months old), accompanied with increased proportion of proinflammatory alveolar macrophages (AMs) that harbor high level of cell surface GRP78 (csGR78) in the AM population. The accumulation of csGR78^high^ AMs is a result of insufficient apoptosis due to ISM1 deficiency (Lam et al. 2022). Hence, ISM1 seems to be a lung resident anti-inflammatory protein important for restraining local inflammation and maintaining lung homeostasis.

Notably, ISM1 expression is upregulated in both bronchial and alveolar epithelial cells at 24 h after intratracheal LPS instillation. In addition, systemic infusion of an antibody against GRP78 significantly attenuated the pulmonary hyperpermeability induced by LPS (Venugopal et al. 2015). These results suggest that ISM1 may affect lung inflammatory response to LPS and ALI.

In this study, we investigated how ISM1 influences LPS-induced acute pulmonary inflammation using *Ism1*^*−/−*^ mice and intranasal instillation of recombinant ISM1 (rISM1). Our data indicated that ISM1 functions as a lung resident inflammation suppressor, protecting the lung from excessive inflammatory responses during LPS-induced ALI and suppresses post-ALI lung fibrosis, likely by suppressing LPS triggered NF-κB activation and proinflammatory cytokine/chemokine production.

## Material and methods

### Mice

ISM1-deficient (*Ism1*^*−/−*^) mice were generated using the CRISPR-Cas9 approach as described in Lam et al. (2022)*.* Adult (7- to 8-week-old) female *Ism1*^*−/−*^ mice in C57BL/6 J background were used in this study. Age- and sex-matched wild-type C57BL/6 J mice were obtained from InVivos Pte. Ltd in Singapore.

### Recombinant proteins

Recombinant ISM1 (rISM1) was mainly produced in *E. coli* BL21DE3 in pET-M bacterial expression vector as a His-tagged protein and purified using Ni–NTA affinity chromatography followed by reverse-phase HPLC as previously described (Zhang et al. 2011; Xiang et al. 2011; Chen et al. 2014). The recombinant protein was confirmed to be endotoxin free (< 1 EU/mg) as determined using Limulus Amebocyte Lysate (LAL) Pyrogen Plus kit (Lonza, N284-25). In rescue experiment, rISM1 was dissolved in filtered PBS, 50 µg of rISM1 per mouse was intranasally administered into the lung. His-tagged rISM1 used in cell culture study was produced from Expi293 cells via transient transfection of pSecTag based mammalian expression plasmid and purified similarly.

### Intratracheal instillation

Mice were anaesthetized with 5% isoflurane (Baxter), followed by intratracheal delivery of LPS (2 mg/kg LPS) from *E. coli* O111:B4 (L2630; Sigma Aldrich) or saline as previously described by Liao et al. (Liao et al. 2008). Control animals received saline alone. The mice were allowed to recover until the time of bronchoalveolar lavage (BAL) collection on day 1, 3, 5 and 7 post LPS instillation for subsequent analysis.

### Bronchoalveolar lavage fluid (BALF) collection

Freshly euthanized mice were dissected to expose the lungs and heart. The tracheas were cannulated and the lungs were lavaged two times with 1 ml of ice-cold PBS. BALF sample was centrifuged at 500 × g for 5 min at 4^*◦*^C. The supernatants were collected and stored at *-*80^*◦*^C till use. Bradford’s reagent was used to quantify BAL protein. 1 mL of erythrocyte lysis buffer (BioLegend 420,301) was added to the cell pellet to lyse all red blood cells, followed by a centrifugation. The live cells were recovered in FACS buffer and counted using Nucleocounter NC-100 (Chemometec, Denmark) or manually counted by hemocytometer followed by differential immune cell count.

### Differential immune cell count

The differential immune cell count was determined by using either NovoCyte or Beckman Coulter CytoFLEX flow cytometers and analyzed with NovoExpress (Acea Biosciences, USA) or CytExpert software (Beckman Coulter, USA). Differential immune cell counts were performed as previously described (Chan et al. 2016). Leukocytes were identified as CD45^+^, alveolar macrophages as CD11c^+^Siglec-F^+^, eosinophils as CD11c^−^Siglec-F^+^, neutrophils as GR-1^+^CD11b^+^ or Ly-6G^+^CD11b^+^, B cells as CD3^−^/CD19^+^ and T cells as CD3^+^/CD19^−^ cells. All antibodies were from Miltenyi Biotech: CD45-VioBlue (130–110-802), CD45-PE (130–110-797), CD64-APC-Vio770 (130–118-685), Siglec-F-PE-Vio770 (130–112-334), Siglec-F-APC (130–102-241), CD11b-PE-Vio615 (130–113-807), CD11b-FITC (130–098-085), CD11c-PE-PerCP700 (130–110-842), CD11c-PE (130–102-799), Ly-6G-PerCP700 (130–117-500), Gr-1-PE (130–112-306), Gr-1-APC (130–102-833), CD3-APC-Vio770 (130–119-793), CD19-PE-Vio770 (130–111-885), Annexin-V-PE (130–118-363), and Viobility 405/520 Fixable Dye (130–109-814).

### Peripheral blood leukocyte counts

Blood was collected from the submandibular vein of the anaesthetized mice. Fresh blood samples were analyzed using Hemavet H950FS Hematology Analyzer (Drew Scientific Group, USA).

### Histology

Fully inflated lungs were fixed in 10% neutral buffered formalin, followed by paraffin embedding, sectioning and staining with hematoxylin and eosin or Picro-Sirius red staining (ab150681, Abcam, USA). Sections (5 µm) were de-paraffinized in histoclear followed by a slow rehydration in a series of alcohol grades starting from 100% ethanol. After hydration to water, the sections were placed in PBS for subsequent staining.

### Immunohistochemistry (IHC) and Immunofluorescence (IF)

Tissue sections were stained with anti-CD68 (sc-7084, Santa Cruz Biotechnology), anti-NIMP-R14 (sc-59338, Santa Cruz Biotechnology), anti-α-SMA (Santa Cruz Biotechnology), anti-TGF-β (sc-146, Santa Cruz Biotechnology), anti-SP-C (sc-13979, Santa Cruz Biotechnology), anti-p65 NF-κB (107,450–1-AP, Proteintech) and anti-PCNA (sc-56, Santa Cruz Biotechnology) overnight at 4 °C. Tissue sections were stained with Hematoxylin and Eosin (DAKO). All images were obtained using Zeiss Axio Imager M2 microscope.

### Immunoblotting and protein array

Fresh tissues were homogenized and centrifuged and soluble supernatants were taken as whole tissue lysates. Standard Western blots were performed using β-actin as the loading control. Antibodies used were anti-TGF-β (sc-146, Santa Cruz Biotechnology), anti-TNF-α (107,590–1-AP, Proteintech), anti-p65NF-κB (10,745–1-AP, Proteintech), anti-P-p65-S276 (AP0123, Abclonal) and anti-P-p65-S536 (AP0475, Abclonal), all used according to manufacturer recommended dilutions. For Western blot, 10–15 µg of whole lung tissue lysate per sample was used. The relative abundance of a variety of 40 cytokines were examined using mouse cytokine proteome profiler array (ARY028, R&D Systems). The whole tissue lysates of 4 mice in each group were used. Equal amount of total protein 200 µg per sample was hybridized to the array and compared for relative expression. The relative expression was quantified by measuring the dot blot intensity using Image J software.

### Cell culture and treatment

Mouse alveolar macrophage cell line MH-S was obtained from ATCC (CRL-2019) and cultured in RPMI-1640 media with 10% FBS. MH-S cells were serum-starved for 5 h before treated with LPS (100 ng/ml) together with rISM1 at various doses simultaneously for 30 min. Cells were harvested and whole cell lysate were generated for subsequent analyses.

### Statistical analysis

Data were expressed as standard errors of the mean (± SEM). Statistical significance was determined using Student’s t-test. *P < 0.05; **P < 0.01, n ≥ 3.

## Results

### ISM1 deficiency leads to increased leukocyte infiltration in the lung under ambient air

**Fig. 1:**
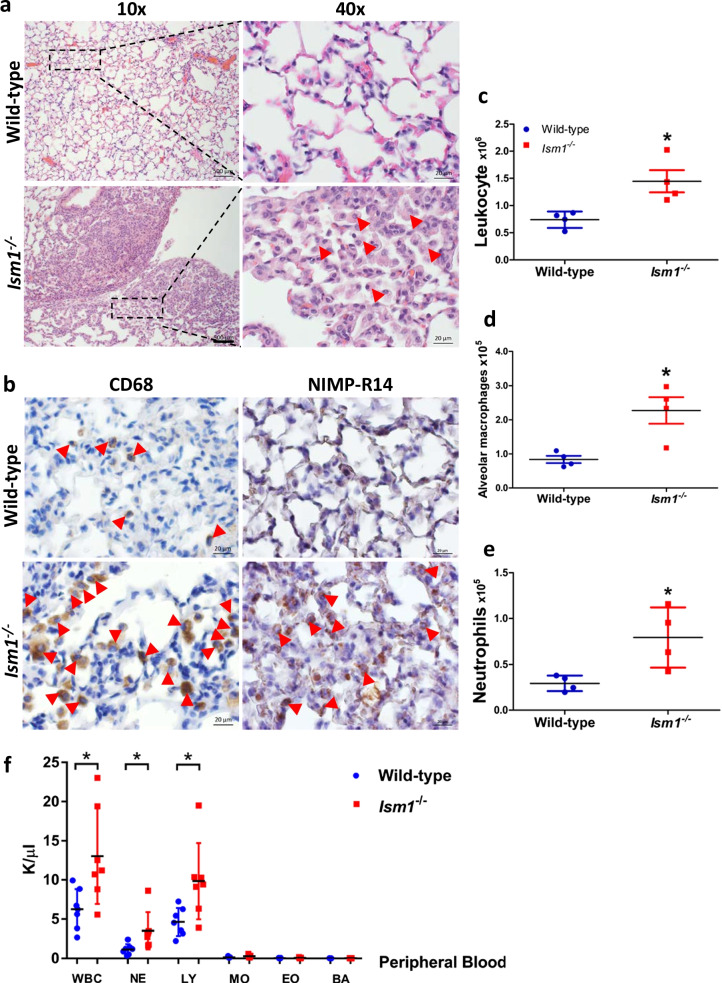
ISM1 deficiency leads to increased leukocyte infiltration in the lung under ambient condition. **a** Increased immune cell infiltration in the lungs of *Ism1*^−/−^ mice at 2 months via H&E staining. **b** Increased macrophages and neutrophils in the lungs of *Ism1*^−/−^ mice at 2 months detected by IHC staining for CD68 and NIMP-R14, respectively. **c**–**e** Differential immune cell count showed increased total leukocytes, alveolar macrophages, and neutrophils in *Ism1*^−/−^ lungs. **f** Analysis of the peripheral blood of *Ism1*^−/−^ mice showed increased total white blood cells, neutrophils and lymphocytes at 2 months. *K/µl* count per µl, *WBC* white blood cell, *NE* neutrophil, *LY* lymphocyte, *MO* monocyte, *EO* eosinophil, *BA* basophil. *represents p < 0.05; **represents p < 0.01. For a-e, n = 4 mice per group; for **f**, n = 7 mice per group

*Ism1* knockout *(Ism1*^*−/−*^*)* C57BL/6 J mice were generated using the CRISPR/Cas9 gene editing method (Lam et al. 2022). *Ism1*^*−/−*^ mice has no obvious developmental abnormalities and is fertile, but exhibited a spontaneous low-grade inflammation in the lung under ambient air. Histological examination of coronal lung sections of 8-weeks old *Ism1*^*−/−*^ mice revealed multi-focal, non-demarcated clusters of inflammatory cells including AMs, polymorphonuclear cells and lymphocytes (Fig. 1a). IHC staining as well as differential immune cell counts of whole lung single-cell homogenates using flow cytometry demonstrated obvious increases in total leukocytes, AMs, and neutrophils (Fig. 1b–e; Additional file 1: Fig. S1). Focal areas of alveolar epithelial hyperplasia, thickening of the alveolar septa as well as emphysema were obvious in *Ism1*^*−/−*^ mice (Fig. 1a). In comparison, *Ism1*^*−/−*^* mice* in FVB/NTac background did not present any increase in neutrophils under ambient environment with no or much milder alveolar epithelial hyperplasia (Lam et al. 2022). As previously reported by us, the spontaneous emphysema phenotype of *Ism1*^*−/−*^ mice is much milder in C57BL/6J strain, accompanied with a milder increase in the pro-inflammatory GRP78^high^ AMs (Lam et al. 2022). Nevertheless, the AM morphological changes in *Ism1*^*−/−*^ lung (increase in large cells and multi-nucleated cells) are shared in both mouse strains. Meanwhile, peripheral blood profiling of *Ism1*^*−/−*^* mice* showed an obvious increase in total white blood cell number compared to that of wild-type mice. Amongst the subpopulations of the peripheral blood white blood cells, lymphocyte and neutrophil numbers were higher in *Ism1*^*−/−*^ mice, while other cell types remain low in both *Ism1*^*−/−*^ and wild-type mice (Fig. 1f). These phenotypes reveal a spontaneous and chronically inflamed lung in conjunction with a mild systemic inflammation when *Ism1* gene is inactivated in C57BL6/J mice. Nevertheless, no obvious distraught or other behavioral changes were observed in *Ism1*^*−/−*^* mice*.

### ISM1 deficiency leads to heightened acute inflammatory response to LPS in the lung

**Fig. 2:**
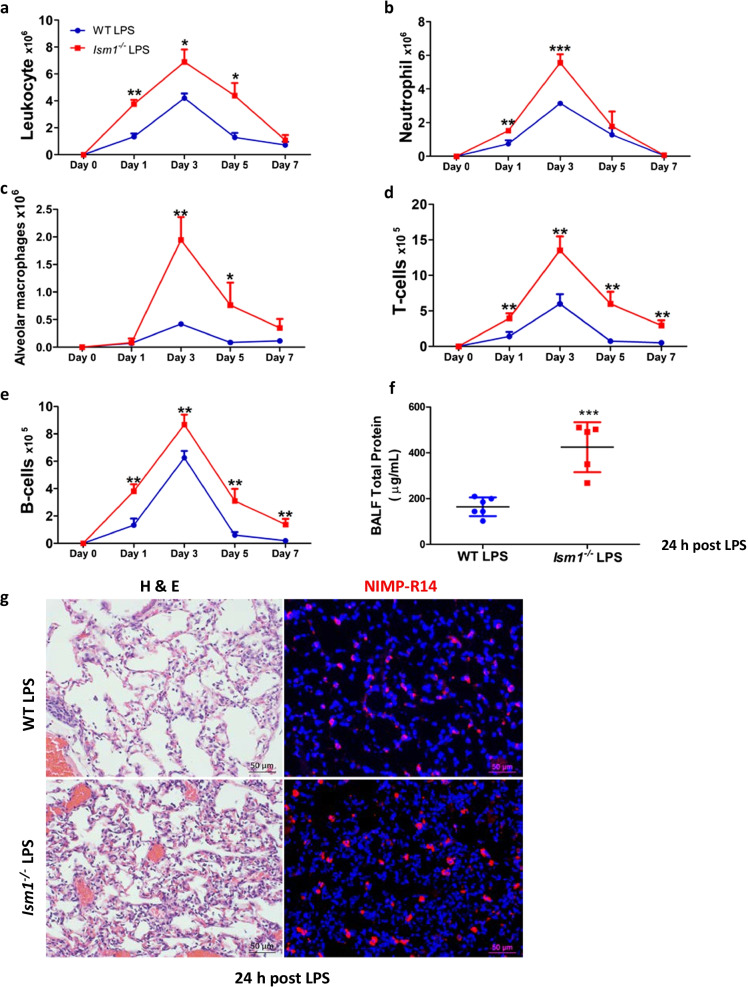
ISM1 deficiency leads to heightened immune responses to LPS in mouse lung*.* **a**–**e** Time-course of lung immune response to LPS challenge. *Ism1*^−/−^ lungs showed heightened immune responses to LPS via differential cell count by flow cytometry. LPS (2 mg/kg) was intratracheally administered once, followed by isolation of single-cell suspension from the whole lungs at day 1, 3, 5 and 7. **f** LPS induced higher pulmonary permeability in *Ism1*^−/−^ mice at 24 h post LPS challenge indicated by total BALF protein content. **g** Representative images of lung tissue sections displaying the extent of the acute inflammation in H&E stained (left) and IF stained NIMP-R14^+^ neutrophils (right) at 24 h post LPS challenge. *represents p < 0.05; **represents p < 0.01. For (**a**–**e** and **g**), n = 3 mice per group; for (**f**), n = 6 for WT and n = 5 for *Ism1*^−/−^ mice

As ISM1 deficiency results in lung inflammation and emphysema and ISM1 expression in the lung is upregulated by intratracheal LPS instillation (Lam et al. 2022; Venugopal et al. 2015), we wish to examine the role of ISM1 in acute lung inflammation in response to intratracheally instilled LPS (2 mg/kg) using *Ism1*^*−/−*^ and wild-type mice. Both groups of mice survived and generated acute inflammatory responses to LPS. Comparing with wild-type mice, *Ism1*^*−/−*^ mice showed a significant increase in total lung leukocytes during the 7-day acute response period (Fig. 2a, Additional file 1: Fig. S2). Higher numbers of neutrophils (Fig. 2b) were observed from day 1 post-LPS challenge in *Ism1*^*−/−*^ mice, while increased AMs in *Ism1*^*−/−*^ mice become prominent from day 3 (Fig. 2c). Both T-cells (Fig. 2d) and B-cells (Fig. 2e) were also significantly increased in *Ism1*^*−/−*^ lung. By day 7, neutrophils have subsided to basal level in both wild-type and *Ism1*^*−/−*^ mice, but an obvious albeit small increase in the number of AMs, T and B cells remain in *Ism1*^*−/−*^ lung, although the differences in AMs are not statistically significant*.* By day 9 post-LPS challenge, the numbers of AMs and B cells in *Ism1*^*−/−*^ lung have also subsided to basal level, but a slightly higher number of T cells remain in *Ism1*^*−/−*^ lung (Additional file 1: Fig. S3). Although ISM1 deficiency under normal ambient condition did not alter mouse lung vascular permeability as shown by Evans blue vascular permeability assay (Additional file 1: Fig. S4), at 24 h post-LPS challenge, a higher vascular permeability was observed in *Ism1*^*−/−*^* mice* by Evans blue assay as well as total protein measurement in bronchoalveolar lavage fluid (BALF), reflecting a heightened lung vascular permeability associated with LPS-induced ALI when ISM1 is absent (Fig. 2f) (Todd et al. 2012). Correspondingly, histology analysis of lungs harvested on day 1 post-LPS challenge (24 h post-LPS) showed a significantly increased immune cells in the alveolar space of *Ism1*^*−/−*^ lungs compared with those of the wild-type lungs, with a noticeable increase of neutrophils (marked by NIMP-R14 staining) (Fig. 2g). Hence, ISM1 deficiency in lung leads to a heightened inflammatory response to intratracheal LPS challenge in mice, supporting an anti-inflammatory role of ISM1 in lung.

**Fig. 3:**
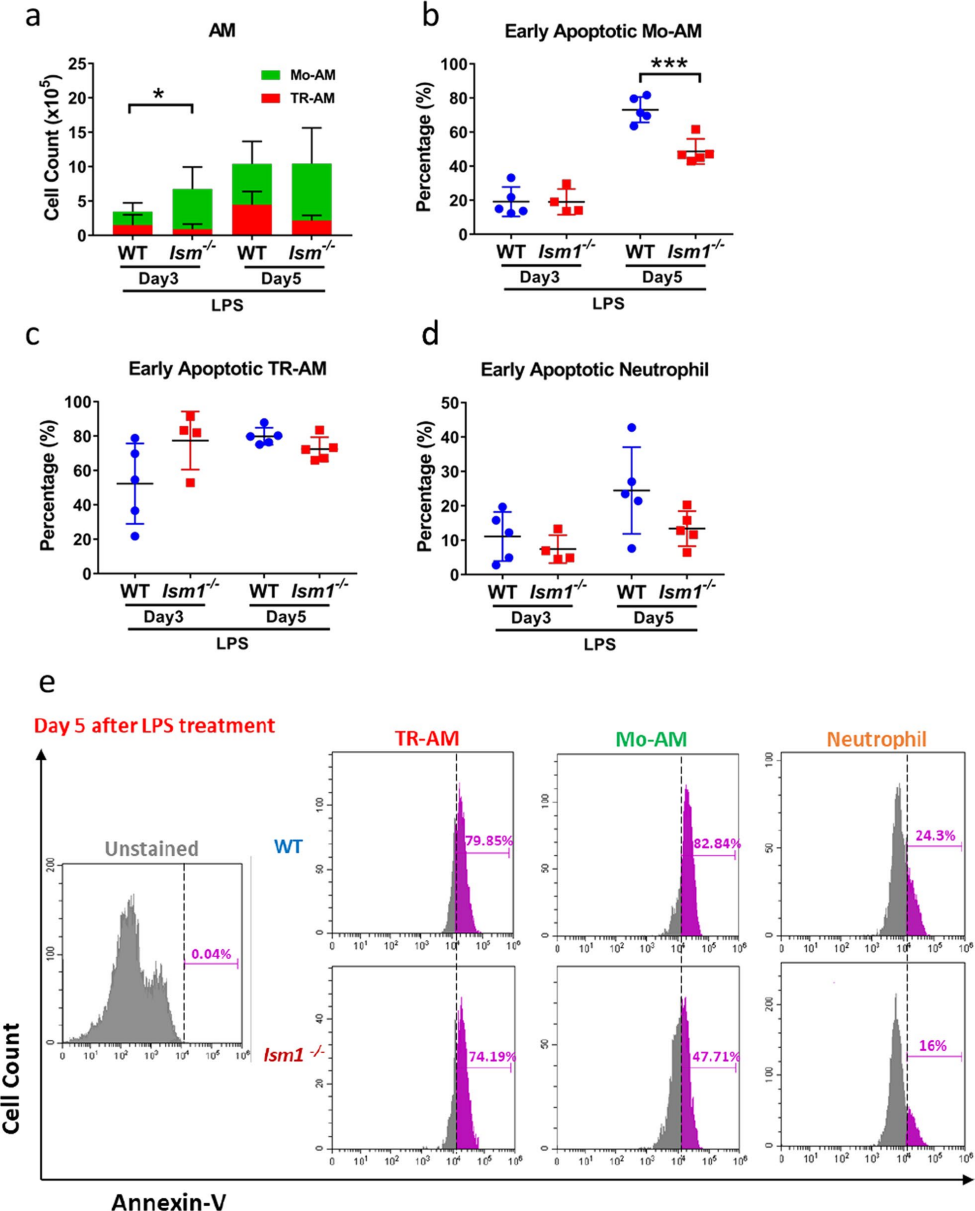
Increase of AMs in *Ism1*^*−/−*^ mouse under LPS is mainly due to increase of Mo-AMs. BALF of 8–9 weeks old WT and *Ism1*^*−/−*^ mice was collected at day 3 and day 5 after intratracheal administration of 2 mg/kg LPS (n = 4–5). After BALF collection, cells were isolated, erythrocytes were lysed, and stained with Viobility fixable dye for live/dead cells, various marker antibodies and Annexin-V and analyzed by flow cytometry. Total cells from BALF were presented in panel a-d. The flow cytometry gating strategy is shown in Additional file 1: Fig. S5. **a** Quantification of the amount of TR-AM and Mo-AM. **b** Percentage of early apoptotic Mo-AM as identified as Annexin-V^+^ in Mo-AM population. **c** Percentage of early apoptotic TR-AM as identified as Annexin-V^+^ cells in TR-AM population. **d** Percentage of early apoptotic neutrophils as identified as Annexin-V^+^ cells in neutrophil population. **e** Representative histograms showing the percentage of Annexin-V^+^ cells in Mo-AM, RT-AM, and neutrophil population on day 5 post LPS. Statistical analysis was conducted by Student's t-test. “*” represents P < 0.05; “***” represents P < 0.001

Further analyses of BALF cells on day 3 and day 5 post LPS-stimulation revealed that the increase in AMs in *Ism1*^*−/−*^ mice is mainly attributed to the increase of monocyte-derived AMs (Mo-AMs) which have migrated to the lung from blood circulation upon LPS stimulation (Fig. 3 and Additional file 1: Fig. S5). In comparison, no significant increase in lung tissue resident AMs (TR-AMs) were observed (Fig. 3a). As ISM1 is a pro-apoptotic protein, we further analyzed BALF immune cell apoptosis using Annexin-V staining, which marks cells in early apoptotic stage. As shown in Fig. 3, on day 5 post LPS instillation when inflammation resolution is already ongoing, apoptosis of Mo-AMs was significantly reduced in *Ism1*^*−/−*^ mice (Fig. 3b). Meanwhile, no significant reduction in the apoptosis of TR-AMs and neutrophils were observed, although there is also an obvious trend in apoptosis reduction in neutrophils on both day 3 and day 5 post LPS instillation (Fig. 3c & d). It should be noted that neutrophils are the first cells to arrive at the inflammatory site and they are short lived. Dead neutrophils would not be detected by Annexin-V staining as we only analyzed live cells. End stage apoptotic cells are presumably quickly phagocytosed and cleared by AMs, since we have previously demonstrated that AMs in *Ism1*^*−/−*^ mice have normal efferocytosis function (Lam et al. 2022). A representative flow cytometry histogram of the Annexin-V^+^ cells were shown in Fig. 3e, presenting the significantly reduced Annexin-V^+^ Mo-AMs in Ism1^−/−^ mice on day 5 post LPS treatment. Hence, increased Mo-AM migration to the lung and reduced apoptosis in Mo-AMs together may have contributed to the higher numbers of AMs in Ism1^−/−^ mice under LPS-triggered acute lung inflammation. It should be noted that apoptotic cells that were in the end stage of apoptosis or necrotic cells are not detected by Annexin-V analyses as we only analyzed Annexin-V^+^ cells in the live cell population.

### Exogenous rISM1 suppresses LPS-induced lung inflammation

**Fig. 4:**
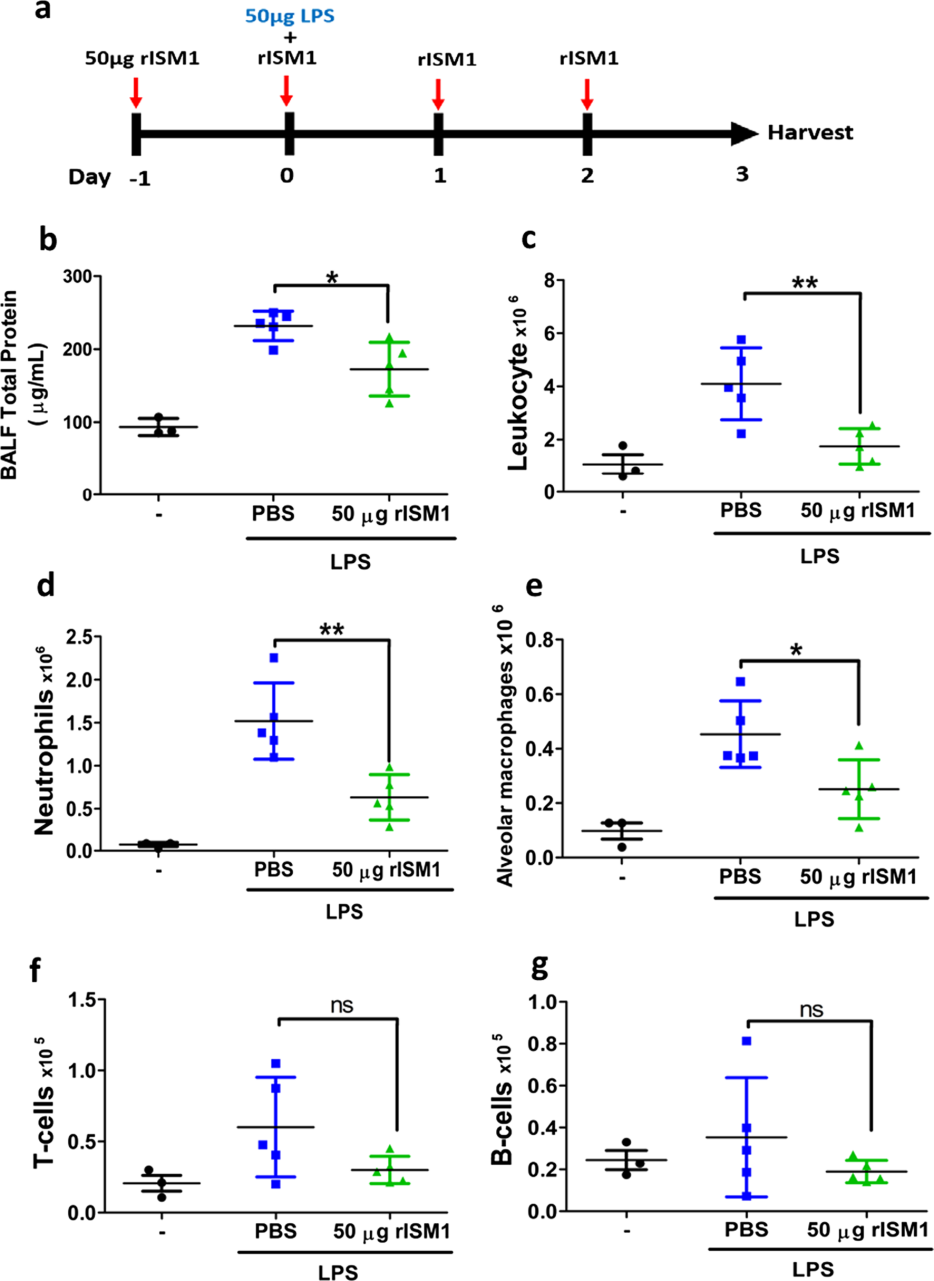
Intranasal rISM1 reduces LPS-induced inflammatory responses in mouse lung. **a** A schematic diagram of rISM1 treatment regimen in intratracheal LPS-treated mice. Each mouse was pre-treated with 50 µg rISM1 via intranasal delivery one day before receiving one dose of 2 mg/kg LPS. The mouse was continuously treated with 50 µg rISM1 daily till day 3. BALF was isolated and separated into the BAL fluid protein and cell components. **b** BALF protein levels were reduced in rISM1-treated mice at 24 h post LPS challenge. Proteins were measured by Bradford protein dye. **c**–**g** Differential immune cell quantification of BALF cells using flow cytometry. Total cells in BALF are presented. rISM1 suppressed immune cell infiltration induced by LPS in the lung. *represents p < 0.05; ** represents p < 0.01. For **b**–**g**, n = 5 mice for LPS-treated groups; n = 3 for non-LPS-treated groups

To further investigate if ISM1 could suppress LPS-induced lung inflammation, we pre-treated wild-type mice with 50 µg rISM1 via intranasal route one day before the single dose LPS intratracheal instillation. rISM1 treatment was continued on the day of and after LPS instillation for three more days once a day (Fig. 4a). BALFs were then collected and rISM1 treated mice indeed showed an obvious reduction in total BALF protein (Fig. 4b). Notably, infiltration of total leukocytes into the alveolar spaces was reduced to almost the basal level by rISM1 treatment at 3 days post-LPS challenge (Fig. 4c). Both neutrophils (Fig. 4d) and AMs (Fig. 4e) were significantly reduced under rISM1 treatment. Although decreases in T and B cells were also observed under rISM1 treatment, these were not statistically significant due to the large variations of cell numbers in the PBS-treated LPS challenged mice (Fig. 4, f & g). Together, these findings support that ISM1 functions as a pulmonary inflammatory suppressor, and local airway delivered rISM1 can quench LPS-induced pulmonary inflammation in mice by reducing LPS-induced neutrophil and macrophage infiltration into the lung.

### ISM1 deficiency leads to defective lung repair and worsened fibrosis post ALI

**Fig. 5:**
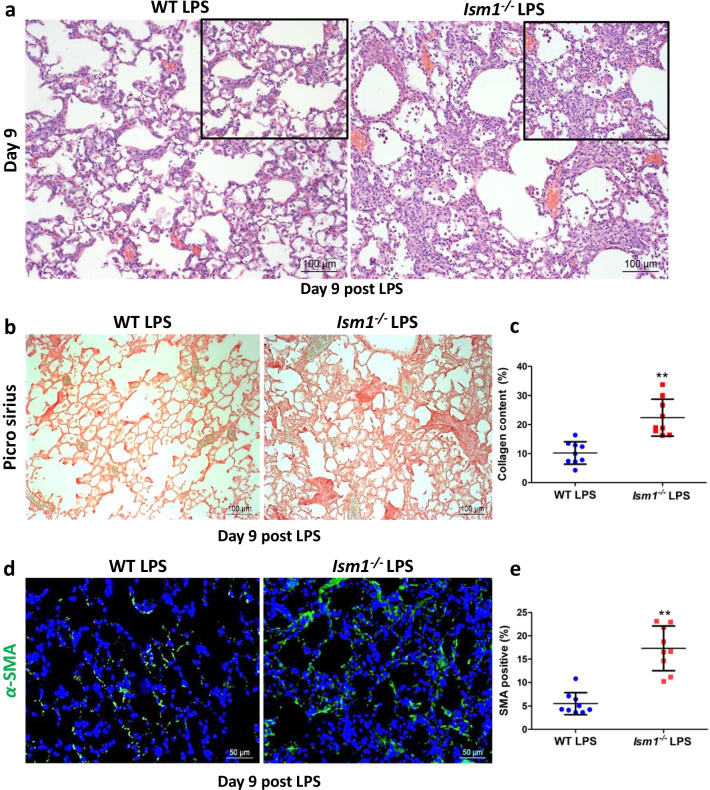
ISM1 deficiency enhances lung fibrosis post LPS-triggered ALI. **a** Representative images of H & E stained lung tissue sections showing the extent of fibrosis in wild-type (WT) and *Ism1*^−/−^ mice at day 9 post LPS challenge. **b**, **c** Collagen deposition, detected by Picro-sirius staining, was higher in the lungs of *Ism1*^−/−^ mice as compared to those of WT mice. **d**, **e** Increased presence of contractile myofibroblasts within fibrotic foci (detected by α-SMA staining) in *Ism1*^−/−^ lungs as compared with those of WT. ** represents p < 0.01. n = 3 mice per group, 2 sections per lung, 5 microscopic fields per section were analyzed

Inflammation is crucial for a proper response to external assaults, yet it can also induce damage to the tissue. The affected tissue will try to repair such inflammation-triggered injuries and restore tissue homeostasis. Excessive inflammatory response can overwhelm the repair mechanism, leading to tissue remodeling. Since *Ism1*^*−/−*^ mice showed a heightened immune response to LPS challenge, we examined whether this heightened inflammatory response would affect lung repair and restoration to homeostasis post ALI. We examined lung tissue histology on day 9 and day 28 post single-dose LPS challenge. As shown in Fig. 5a, *Ism1*^*−/−*^ lung showed severe distortion of lung tissue structure with wide-spread thickening of alveolar wall and alveolar septa. A marked increase in collagen deposition was also observed by Picro-Sirius Red staining (Fig. 5b, c; Additional file 1: Fig. S6a). Moreover, increased abundance of myofibroblasts (*α*-smooth muscle actin positive, α–SMA^+^) is obvious, in particular in small fibrous clusters (Fig. 5d, e). At 28 days post 3-dose LPS challenge, obvious scars were observed in *Ism1*^*−/−*^ lung (Additional file 1: *Fig. S*6*b).* Excessive accumulation of extracellular matrix (ECM), myofibroblasts and scar formation are characteristics of pulmonary fibrosis (Todd et al. 2012).

**Fig. 6:**
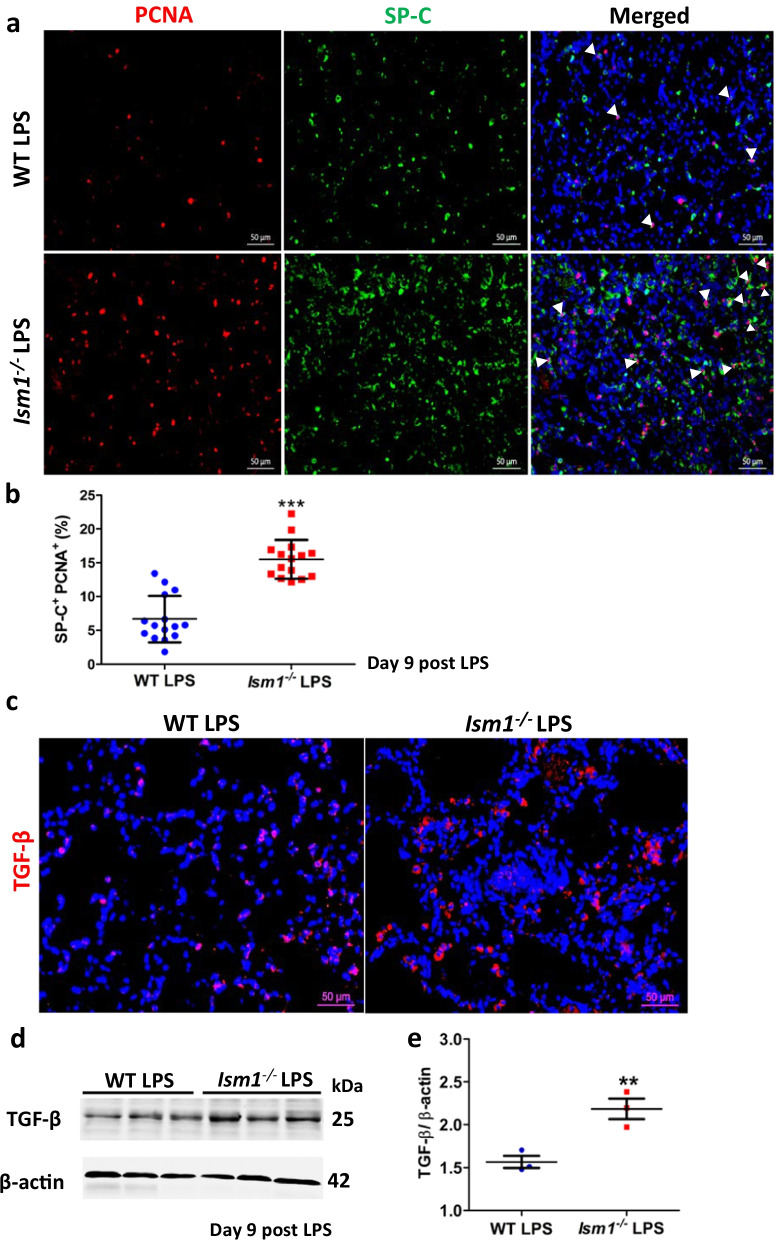
ISM1 deficiency increases proliferation of alveolar epithelial type 2 cells (AT2) and elevates profibrotic TGF-β in mouse lung. **a** Representative images of SP-C and PCNA double IF stained lung sections of *Ism1*^−/−^ and WT mice at day 9 post LPS challenge. **b** Quantification of the percentage of SP-C^+^PCNA^+^ cells in the lung of *Ism1*^−/−^ and WT mice at day 9 post LPS challenge. Increased number of proliferating AT2 cells in the lungs of *Ism1*^−/−^ mice were shown. *** represents p < 0.001. n = 3 mice per group, 2 sections per lung, 5 microscopic fields per section were analyzed. **c** Representative images of IF stained TGF-β lung sections of *Ism1*^−/−^ and WT mice at day 9 post LPS challenge. n = 3 animals per group, 2 sections per lung, 5 microscopic fields per section were analyzed. **d**, **e** TGF-β protein level in the lung of *Ism1*^−/−^ and WT mice at day 9 post LPS challenge by Western blot of whole lung lysate **d** and quantification (**e**). ** represents p < 0.01. n = 3 mice per group, 1 total lung lysate per mouse

LPS-induced lung inflammatory responses induce injury/damage to the alveolar epithelium. There are two types of alveolar epithelial cells: type I (AT1) cells are believed to be terminally differentiated, flat, squamous and covering 90% of alveolar wall surface; type II (AT2) cells are less in number but possess stem cell-like property (Williams 2003). AT2 cells are important for lung repair and regeneration following lung injury through proliferation and differentiation into AT1 cells to re-epithelialize the alveolar walls (Kasper and Barth 2017; Fehrenbach et al. 2000). Aberrant replacement of AT1 cells by hyperplastic AT2 cells is one of the contributing factors to lung fibrosis (Kasper and Barth 2017). At day 9 post-LPS challenge, *Ism1*^*−/−*^ lungs showed significantly more proliferating AT2 cells than those in wild-type lungs as shown by double immunofluorescent staining for PCNA (proliferation marker) and SP-C (AT2 marker) (Fig. 6a, b). This result suggests that AT2 hyper-proliferation in *Ism1*^*−/−*^ lung could be one reason for the heightened fibrosis post-LPS induced ALI.

TGF-*β* is the most potent pro-fibrotic mediator characterized to date (Biernacka et al. 2011). Indeed, at day 9 post-LPS challenge, TGF-*β* level was significantly increased in *Ism1*^*−/−*^ lungs compared with wild-type lungs (Fig. 6c–e).

In addition, in both single-dose and repeated-dose intratracheal LPS challenge experiments, we observed a heightened lung fibrosis at 28 days post first LPS-challenge in *Ism1*^*−/−*^ lung, which are represented by higher collagen deposition and obvious scar structure formation (Additional file 1: Fig. S6) (Li et al. 2009). By this time, all immune cell infiltrations have subsided to basal levels in both wild-type and *Ism1*^*−/−*^ lungs (data not shown), but the lung fibrosis remains.

Altogether, these results indicate that ISM1 deficiency led to a heightened lung inflammatory response to intratracheal delivered LPS, leading to abnormal lung repair and intensified post-ALI fibrosis in mice.

### ISM1 deficiency heightened production of proinflammatory cytokines/chemokines in lung and enhanced LPS-induced NF-κB activation

**Fig. 7:**
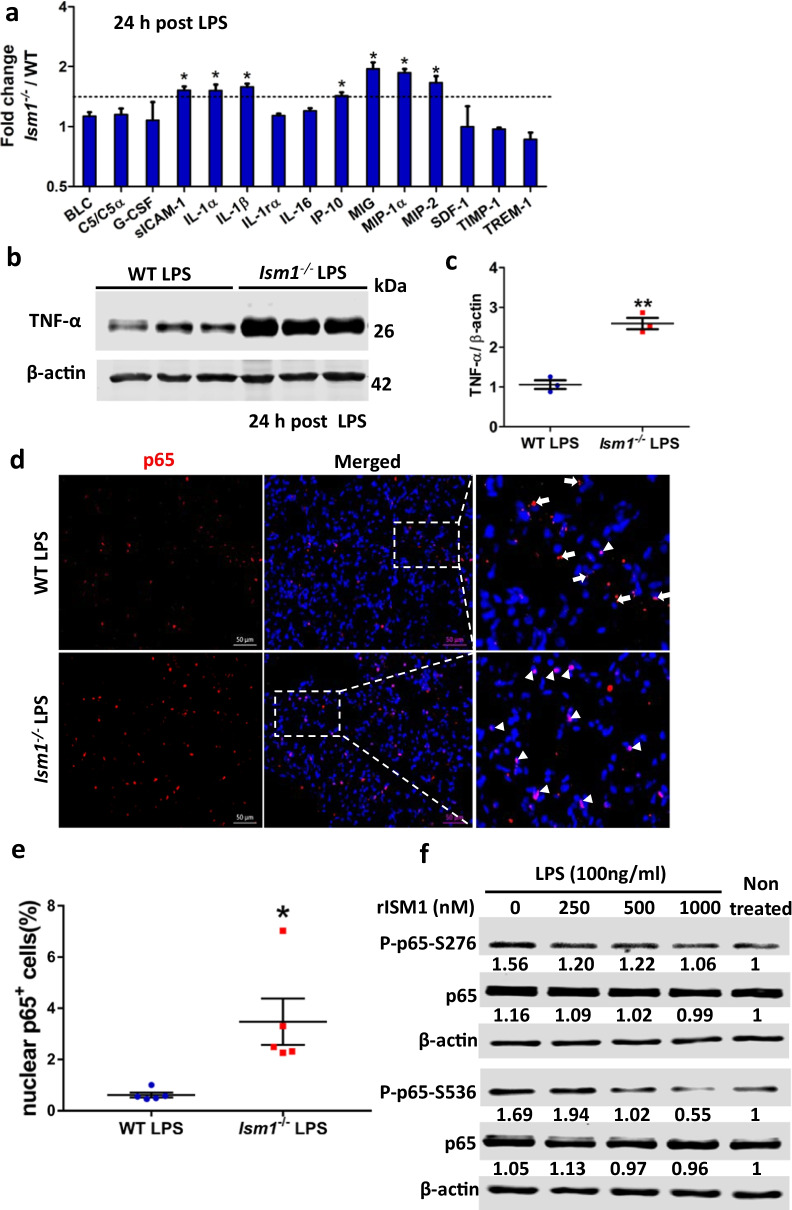
ISM1 deficiency increased proinflammatory cytokine/chemokine production in lung and promoted LPS-induced NF-κB activation. **a** Lung cytokine/chemokine profile determined by an inflammatory cytokine antibody array using lung homogenates at 24 h post LPS challenge. * represents p < 0.05. n = 4 mice per group, 1 total lung lysate per mouse. **b**, **c** Increased levels of TNF-α in *Ism1*^−/−^ lung 24 h post LPS challenge shown by Western blot of whole lung lysate. ** represents p < 0.01. n = 3 mice per group, 1 total lung lysate per mouse. **d**, **e** IF staining showing increased p65 NF-κB nuclear translocation in *Ism1*^−/−^ lung. Representative images showing increased signal co-localization of the p65 NF-κB (red) and the DAPI nucleus (blue) in the lung sections of *Ism1*^−/−^ mice **d** and the quantification of nuclear p65 NF-κB in panel d, showing the percentage of cells with nuclear localized p65 in WT and *Ism1*^−/−^ lung (**e**). n = 5 mice per group, 2 sections per lung, 5 microscopic fields per section. **f** rISM1 dose-dependently suppresses LPS-induced NF-κB p65 phosphorylation at S276 and S536 in cultured mouse MH-S alveolar macrophage cells. Cells are harvested at 30 min post LPS treatment (100 ng/ml). Based on two independent experiments

We further examined if the exaggerated post-LPS lung inflammation in *Ism1*^*−/−*^ lung is due to an altered cytokines/chemokines production. Using a mouse cytokine antibody array and whole lung lysates, we show that at 24 h post-LPS challenge, seven cytokines/chemokines were substantially elevated (≥ 1.5-fold) in *Ism1*^*−/−*^ lung compared with those in wild-type lung (Fig. 7a). All seven up-regulated cytokines/chemokines are known proinflammatory mediators including IL-1*α*, IL-1*β*; leukocyte chemoattractant monokine induced by gamma interferon (MIG), C-X-C chemokine CXCL10/IP-10, macrophage inflammatory protein 1α (MIP-1α, CCL3), macrophage inflammatory protein 2 (MIP-2, CXCL2) as well as soluble ICAM-1 (intercellular adhesion molecule 1). In addition, there was also an almost threefold increase of TNF-*α* in the *Ism1*^*−/−*^ lungs demonstrated by Western blot (Fig. 7b, c). These results indicated that ISM1 deficiency led to increases of multiple proinflammatory cytokines upon LPS challenge, which could contribute to the exaggerated inflammation in *Ism1*^*−/−*^ lung.

It is known that LPS activates NF-κB, the master regulator of inflammation, in murine lungs by inducing the nuclear translocation of NF-κB (Park et al. 2015). To determine whether ISM1 inhibits LPS-induced acute lung inflammation by interfering with LPS-induced NF-κB activation, lungs tissues of *Ism1*^*−/−*^ and wild-type mice at 24 h post-LPS challenge were fixed and stained for NF-κB p65 subunit. As shown in Fig. 7d, e, *Ism1*^*−/−*^ lung tissue sections showed a significant increase in nuclear localized p65 (red) compared with that of the wild-type lung, suggesting an enhanced NF-κB activation. In addition, rISM1 dose-dependently suppressed LPS-induced NF-κB p65 phosphorylation at both S276 and S536 in cultured MH-S mouse AMs (Fig. 7f). These data revealed that another anti-inflammatory mechanism of ISM1 in mouse lung could be its ability to suppress LPS-induced NF-kB activation in AMs.

## Discussion

Using LPS-induced ALI model, we have demonstrated here that ISM1 deficiency leads to heightened acute inflammatory response in the mouse lung. Furthermore, intranasal instillation of rISM1 could quench LPS-induced acute lung inflammation in mice. ISM1 deficiency also lead to intensified post-ALI pulmonary fibrosis in mice. These findings revealed that endogenous ISM1 has a protective function in mouse lung by restraining the level of inflammatory response under LPS challenge and facilitating the post-ALI lung to repair and regain homeostasis.

Although *Ism1*^*−/−*^ lungs exhibited more severe inflammatory response to LPS, the temporal activation of immune response is similar to that of wild-type mice, peaking on day 3 and thereafter gradually subsides, almost reaching the basal level on day 7 (Fig. 2). We showed that the heightened increase in AMs under LPS-triggered acute lung inflammation in *Ism1*^*−/−*^ mice are mainly due to the increase in Mo-AMs that migrated to the lung from blood circulation (Fig. 3a). In addition, the significantly reduced Mo-AM apoptosis most likely also contributed to the increased Mo-AMs in *Ism1*^*−/−*^ lung.

Following the acute inflammation of ALI, the lung goes into repair and remodeling, trying to recover and regain hemostasis and restore function (Gill et al. 2017). During the repair and remodeling, fibro-proliferation occurs, which is eventually resolved in wild-type mouse lung. However, *Ism1*^*−/−*^ lungs exhibited prolonged and heightened lung fibrosis and scar formation. Under the single-dose LPS treatment model, at day 9 post-LPS, *Ism1*^*−/−*^ lungs showed more extensive fibrosis-like phenotype with increased collagen deposition, enhanced myofibroblasts accumulation and higher TGF-β expression level, in conjunction with a heightened AT2 proliferation (Figs. 5 and 6). This heightened pulmonary fibrosis correlated with the upregulation of the pro-fibrosis cytokine TGF-β at this stage (Fig. 6c–e). The heightened lung fibrosis retains at least to 28 fays post LPS treatment. Under repeated LPS treatment model, *Ism1*^*−/−*^ lung also presented a higher level of scar formation at 28 days post LPS challenge, indicating persistent tissue remodeling when ISM1 is deficient (Additional file 1: Fig. S6). These data reveal a ‘hyper-responsive’ lung when ISM1 is deficient that responds to LPS with excessive inflammation, a condition that commonly results in tissue remodeling (Matthay et al. 2012).

AT2 cells have critical secretory and regenerative roles in the alveolus to maintain lung homeostasis but they are also drivers of lung fibrosis in idiopathic pulmonary fibrosis (IPF) (Parimon et al. 2020). The lungs of IPF patients are often characterized by the presence of fibroblastic foci surrounded by hyperplastic AT2, which drive persistent tissue remodeling (Parimon et al. 2020; Phan et al. 2021). Hyper-proliferation of AT2 cells compensates for epithelial cell damage in IPF, which leads to epithelial hyperplasia. Our findings here indicate that endogenous ISM1 of the lung has a protective role in restraining the extent of LPS-induced acute lung inflammation, protecting the lung from severe damage and facilitate post-injury repair and recovery. Excessive inflammation ensues in *Ism1*^*−/−*^ lung following LPS assault, which likely overwhelms the lung’s repair mechanism, leading to dysregulated repair, hence heightened and prolonged structural alteration and fibrosis.

ALI/ARDS is marked by a profound presence of activated neutrophils together with fluid accumulation in lung, leading to lung function impairment and high mortality (Ragaller and Richter 2010). ALI/ARDS are prevalent acute pulmonary diseases in human population (Johnson and Matthay 2010). No pharmacological therapeutic breakthroughs have been reported thus far (Matthay et al. 2012). Our findings here that intranasal delivered rISM1 can effectively reduce leukocyte infiltration into the lung upon airway LPS challenge in mice, including both neutrophils and AMs, suggest that rISM1 may be useful in facilitating lung repair and recovery for acute lung damage due to bacterial infection in clinical settings. Importantly, we found that LPS-induced lung hyperpermeability was also mildly reduced by intranasal rISM1 treatment, likely a consequence of inflammation suppression (Fig. 4). These findings are consistent with our recent report that ISM1 deficiency lead to spontaneous inflammation and emphysema in young adult mouse lung, and intratracheal delivered rISM1 could effectively suppress cigarette-smoke induced lung inflammation and restore lung function in a cigarette-smoke induced emphysema model (Lam et al. 2022). Findings from this work further support the notion that ISM1 is a lung resident anti-inflammatory protein in mice.

Previous reports indicated that NF-κB pathway plays a key role in LPS-induced inflammation (Selvaraj et al. 2015; Grossman et al. 2002). The activation of NF-κB results in the translocation of its active form p65 into nucleus. LPS is known to enhance translocation of NF-κB p65 from cytoplasm to nucleus (Wang et al. 2015). LPS-induced NF-κB activation is also known to increase the expression of proinflammatory cytokines such as IL-1, MIP-2 and TNF-α, leading to excessive inflammation response (Shi et al. 1999). We found that in *Ism1*^*−/−*^ lung, NF-κB nuclear translocation is enhanced, correlating with the upregulation of several proinflammatory cytokines/chemokines including IL-1*α*, IL-1*β*, MIG, CXCL10/IP-10, MIP-1α, MIP-2 and TNF-α (Fig. 7b, c). Furthermore, rISM1 dose-dependently suppressed LPS-induced NF-κB activation in cultured mouse AM MH-S cells by suppressing LPS-induced phosphorylation of S276 and S536 (Fig. 7f). Hence, lung resident ISM1 inhibit LPS-induced acute lung inflammation by inhibiting LPS-induced NF-κB activation as well as the production of multiple proinflammatory cytokines.

We have previously reported that intravenous bolus administered rISM1 alone can induce lung vascular permeability in mice from 15 min after injection and this ISM1-induced lung vascular permeability peaked at 1 h post ISM administration and subsided to basal level after 24 h (Venugopal et al. 2015). It seems that the lung vascular permeability inducing activity of rISM1 is transient in vivo and quickly subsided. In this work, we showed that when rISM1 is delivered via intranasal route in a repeated treatment regimen (Fig. 4), it suppressed LPS-induced lung inflammation at 72 h post LPS challenge and 24 h after the last rISM1 instillation. This result is consistent with findings in previous work as well as this work that endogenous ISM1 is an anti-inflammatory protein that functions to protect lung homeostasis (Lam et al. 2022). The different delivery route may explain the differences observed in rISM1’s function in lung vascular permeability under different settings. It is reasonable to speculate that the efficiency of intravenously delivered ISM1 protein (54 kDa) to reach the airway would be extremely low. In contrast, intranasal delivered rISM1 can directly interact and affect airway immune cells as well as bronchial and alveolar epithelial cells. The suppression of LPS-induced vascular permeability by intranasal rISM1 is likely a result of ISM1’s suppression of LPS-induced acute inflammation via inhibiting NF-κB activation as well as suppressing the production of multiple proinflammatory cytokine/chemokine (Fig. 7a–c).

ISM1 was also reported by us as an anti-angiogenic protein (Xiang et al. 2011). Several endogenous angiogenic inhibitor proteins have also been previously reported to suppress pulmonary inflammation in addition to their anti-angiogenic function. For example, thrombospondin-1 (TSP-1) was reported to be critical in physiological inflammation and homeostasis in the mouse lung (Lawler et al. 1998). As early as 1-month old, *Tsp1*^*−/−*^ mice started to show patchy sites of inflammation in their lung parenchyma. Neutrophilic infiltrates manifested in the alveoli and perivascular connective tissue. Similar to *Ism1*^*−/−*^ mice, *Tsp1*^*−/−*^ mice were also more susceptible to LPS-induced lung injury (Zhao et al. 2014). TSP-1 curbs inflammatory responses via regulating the production of IL-10, a key anti-inflammatory cytokine during the resolution phase of lung injury. Angiostatin is another angiogenic inhibitor that has an anti-inflammatory function, suppressing LPS-induced acute lung injury in mice (Chavakis et al. 2004; Aulakh et al. 2014a, 2014b). Treatment of angiostatin via subcutaneous administration was shown to potently reduced protein accumulation in BALF and leukocyte infiltration into the lung within day 1 post LPS-challenge (Aulakh et al. 2014a). In this work, we show that ISM1 functions in similar fashion to TSP-1 since both knockout mice exhibit heightened inflammatory responses in the lung under non-pathological condition; and hyper-responsiveness in LPS-induced acute lung injury. Meanwhile, both exogenous angiostatin (subcutaneous) and ISM1 (intranasal) could suppress acute lung inflammation upon LPS challenge.

A limitation of our study is that rISM1 was given both before and after LPS instillation in our experimental setup. This is different from the clinical reality in which most ALI patients only receive treatment after the lung inflammation has been well established. To better evaluate the therapeutic value of rISM1 in ALI/ARDS, future studies should investigate the efficacy of rISM1 after ALI has already been triggered. Nevertheless, this work revealed ISM1 as a novel lung endogenous protein that plays a role in restraining LPS-triggered acute lung inflammation, ALI and post-ALI pulmonary fibrosis. rISM1 therefore has potential therapeutic value for ALI and pulmonary fibrosis.

## Conclusion

This work demonstrated that ISM1 is a protective factor in mouse lung, restraining the level of inflammatory response in LPS-induced ALI and limiting post-injury pulmonary fibrosis by suppressing LPS-triggered production of pro-inflammatory cytokines and NF-κB activation. rISM1 has the potential to be further developed into a therapeutic for ALI and ALI-linked pulmonary fibrosis.

## Supplementary Information

**Additional file 1. **Additional figures.

## Data Availability

All data presented in the main manuscript and supplementary files are available. Research materials are available from the corresponding author.

## Abbreviations

ISM1: Isthmin 1
LPS: Lipopolysaccharide
ALI: Acute lung injury
ARDS: Acute respiratory distress syndrome
*csGRP78*: Cell surface glucose regulated protein of 78 kDa
TGF-β: Transforming growth factor beta
TNF-α: Tumor necrosis factor α
TLR-4: Toll-like receptor 4
IL-1*α* and IL-1*β*: Interleukin-1α and 1β
MIG: Leukocyte chemoattractant monokine induced by gamma interferon
MIP-1α: Macrophage inflammatory protein 1α
MIP-2: Macrophage inflammatory protein 2
ICAM-1: Intercellular adhesion molecule 1
IHC: Immunohistochemistry
IF: Immunofluorescence
H & E: Hematoxylin and eosin
