# Supplementary material for: ISM1 suppresses LPS-induced acute lung injury and post-injury lung fibrosis in mice
Source: Mol Med. 2022 Jun 25;28:72. doi: 10.1186/s10020-022-00500-w (PMC9233842; doi:10.1186/s10020-022-00500-w)
Supplement: Supplementary file 1 — Additional file 1. Additional figures. [file 10020_2022_500_MOESM1_ESM.pptx]

## Slide 1
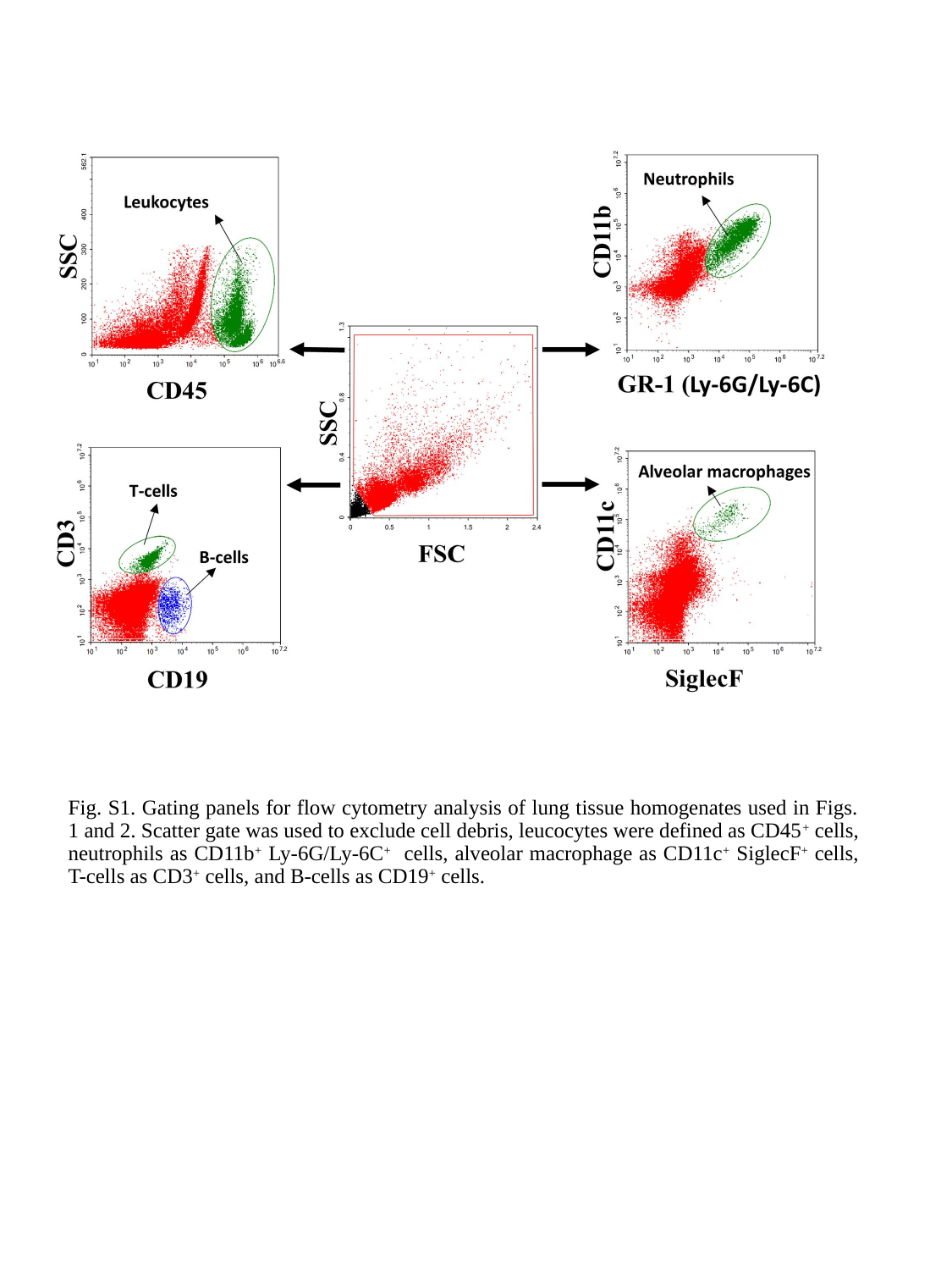

# Fig. S1. Gating panels for flow cytometry analysis of lung tissue homogenates used in Figs. 1 and 2. Scatter gate was used to exclude cell debris, leucocytes were defined as CD45+ cells, neutrophils as CD11b+ Ly-6G/Ly-6C+ cells, alveolar macrophage as CD11c+ SiglecF+ cells, T-cells as CD3+ cells, and B-cells as CD19+ cells.

## Slide 2
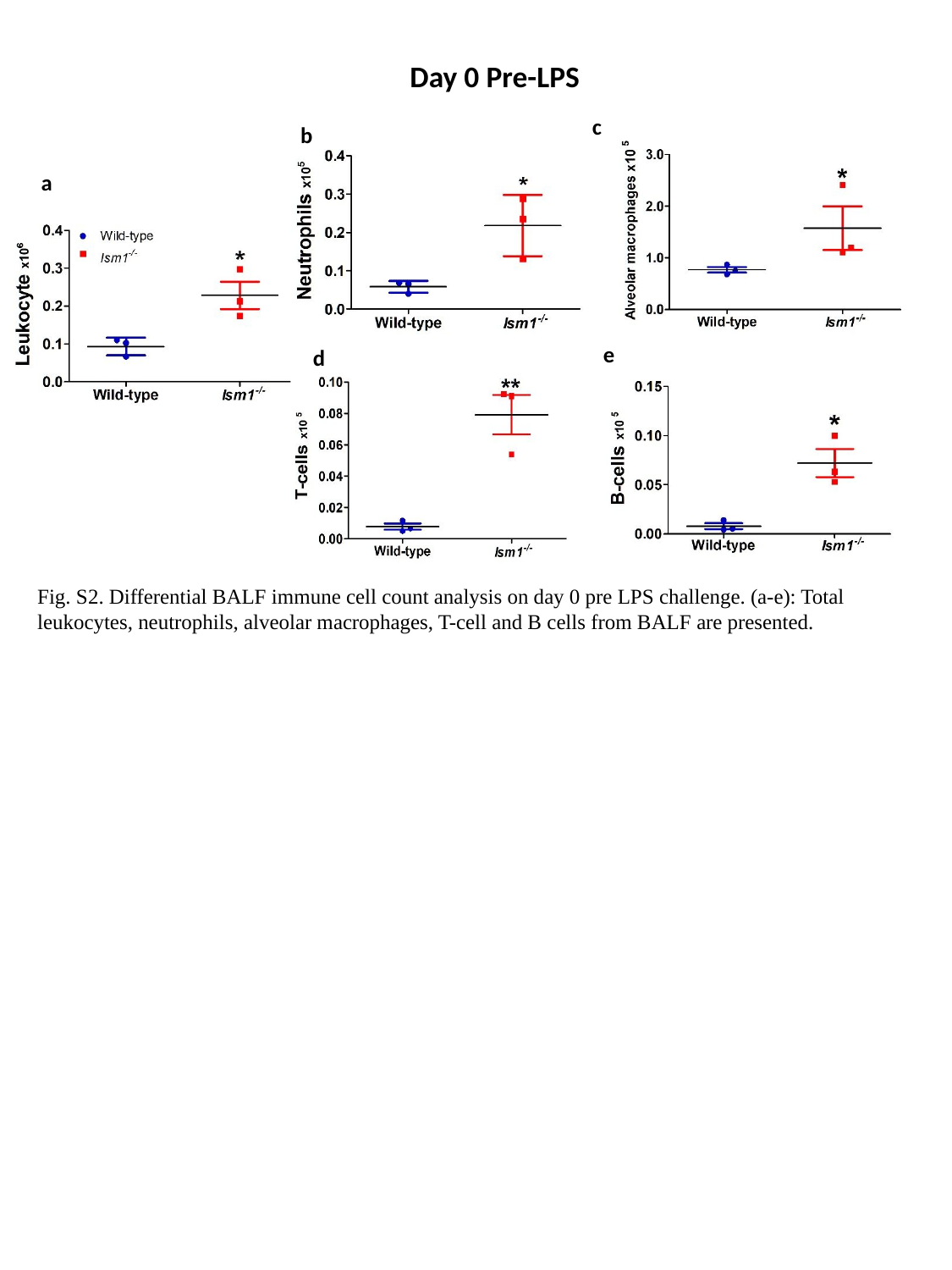

Day 0 Pre-LPS
c
b
a
e
d
Fig. S2. Differential BALF immune cell count analysis on day 0 pre LPS challenge. (a-e): Total leukocytes, neutrophils, alveolar macrophages, T-cell and B cells from BALF are presented.

## Slide 3
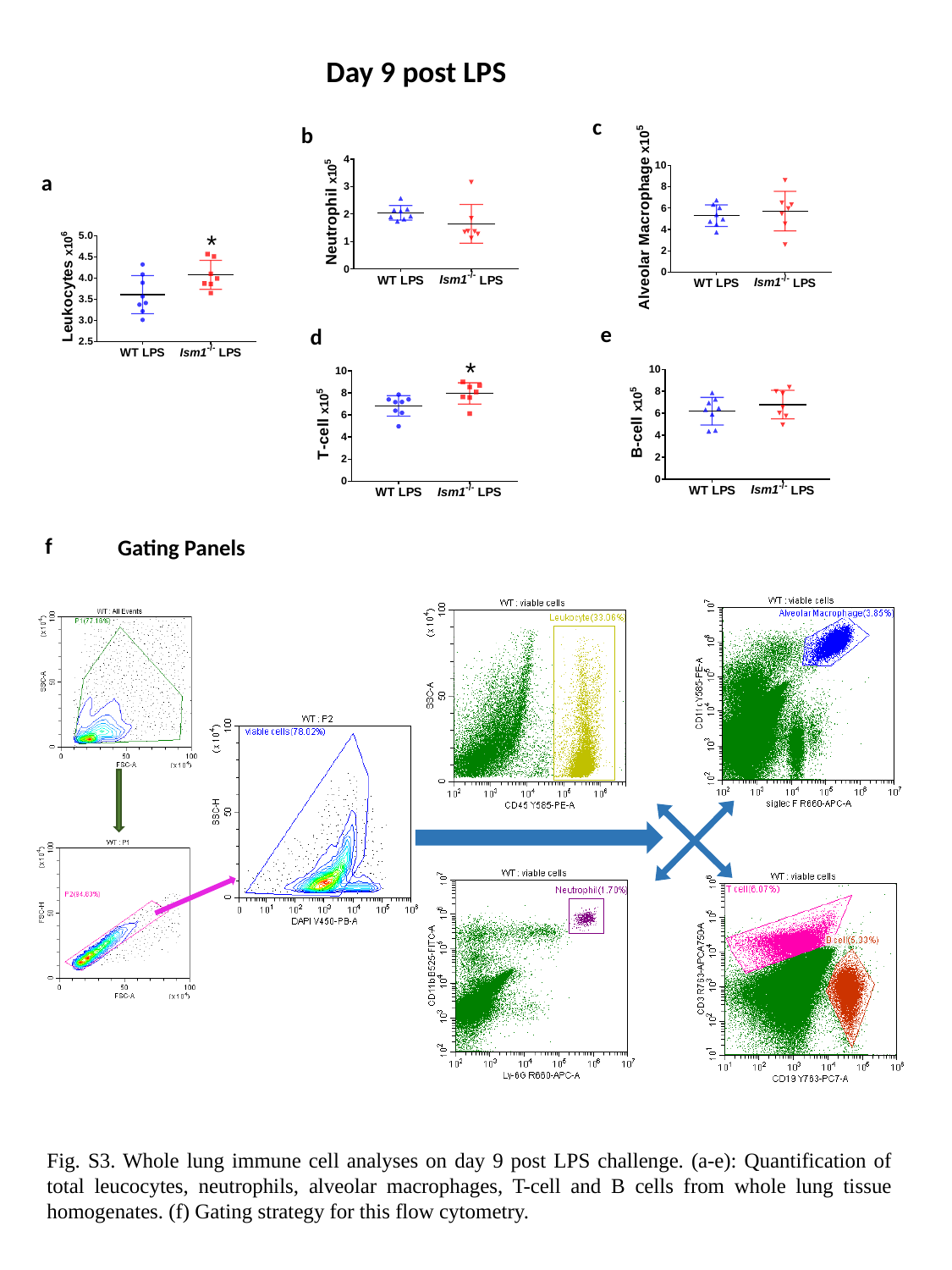

Day 9 post LPS
c
b
a
e
d
f
Gating Panels
Fig. S3. Whole lung immune cell analyses on day 9 post LPS challenge. (a-e): Quantification of total leucocytes, neutrophils, alveolar macrophages, T-cell and B cells from whole lung tissue homogenates. (f) Gating strategy for this flow cytometry.

## Slide 4
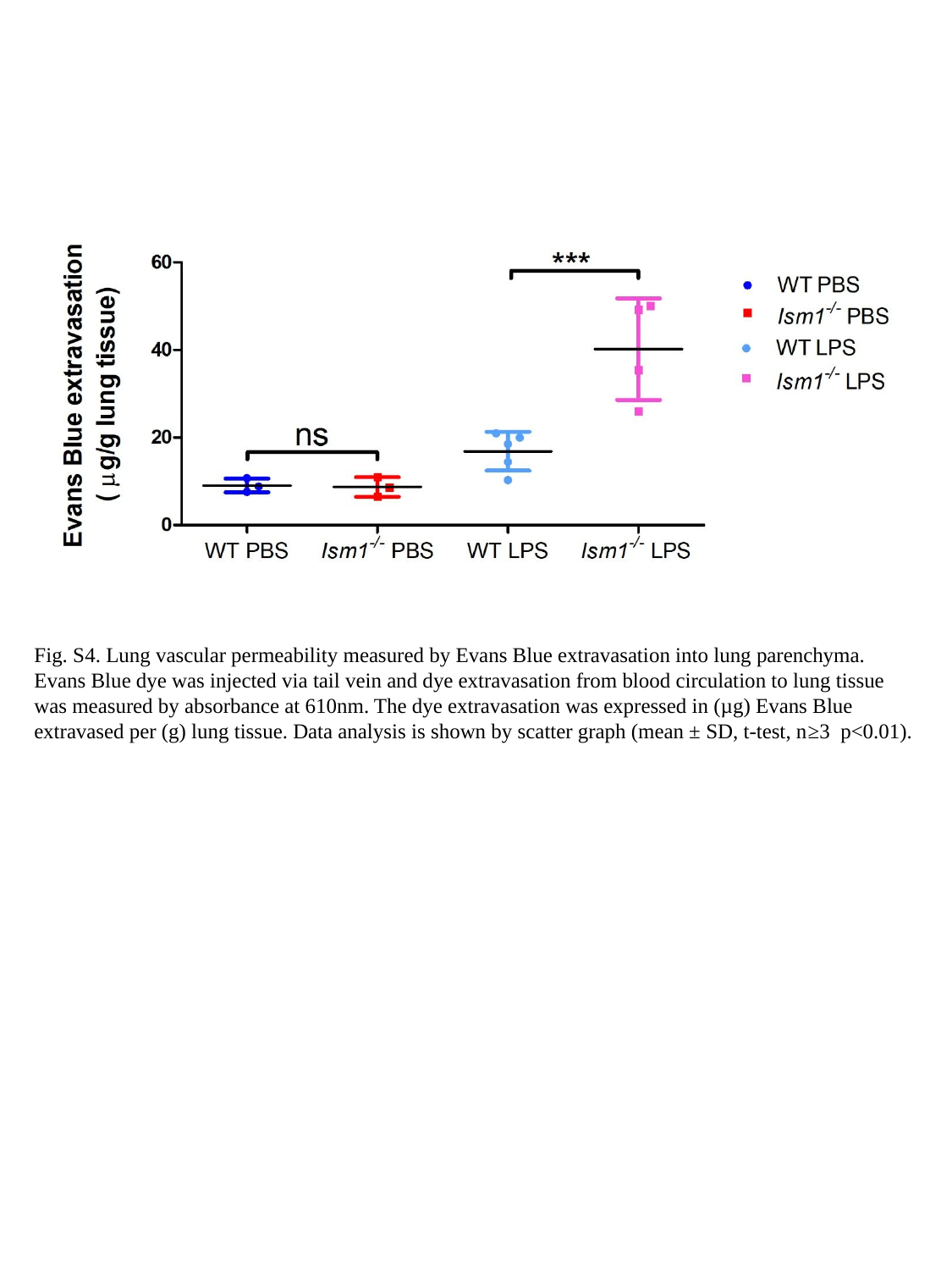

Fig. S4. Lung vascular permeability measured by Evans Blue extravasation into lung parenchyma.
Evans Blue dye was injected via tail vein and dye extravasation from blood circulation to lung tissue
was measured by absorbance at 610nm. The dye extravasation was expressed in (µg) Evans Blue extravased per (g) lung tissue. Data analysis is shown by scatter graph (mean ± SD, t-test, n≥3 p<0.01).

## Slide 5
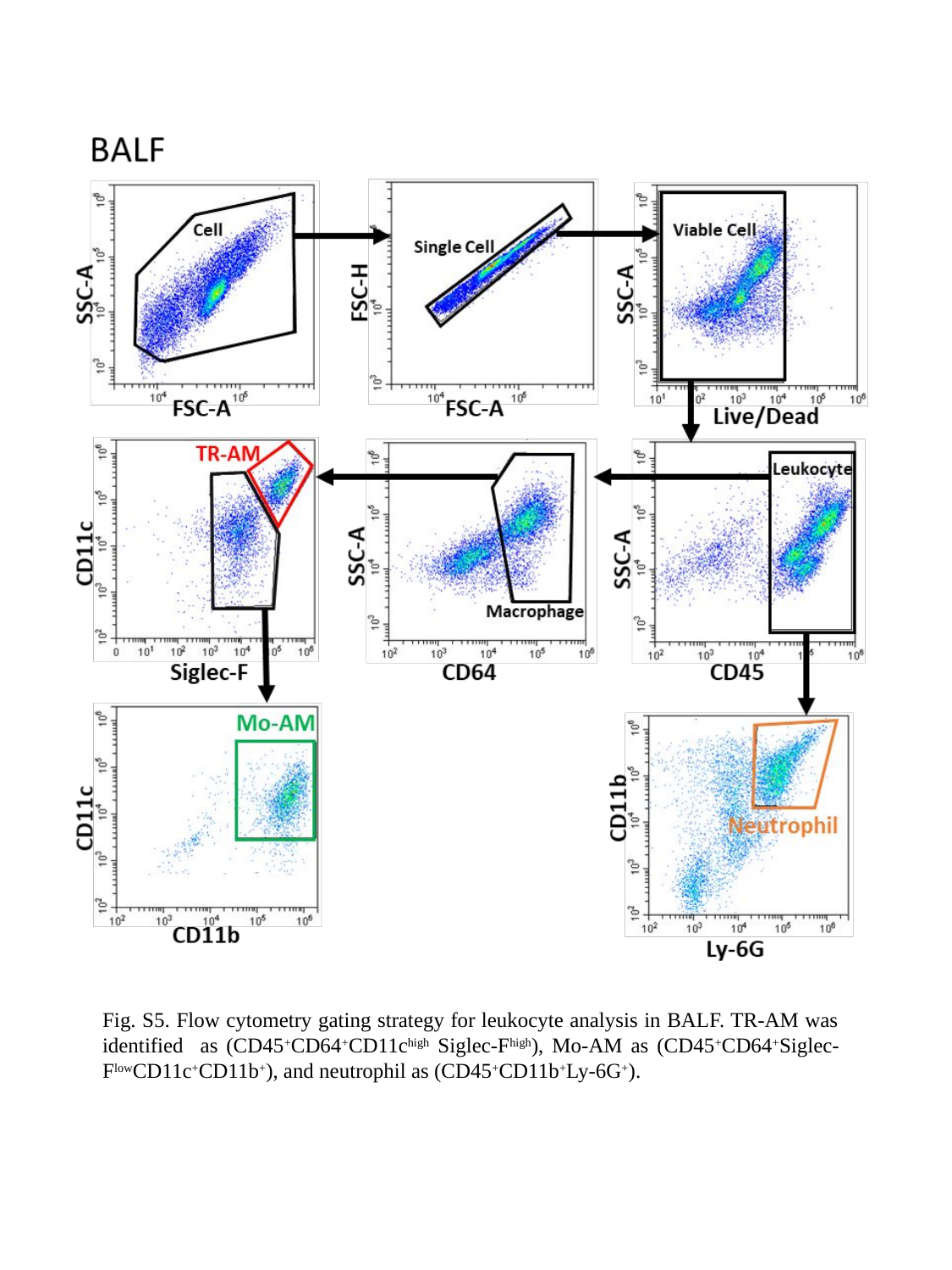

Fig. S5. Flow cytometry gating strategy for leukocyte analysis in BALF. TR-AM was identified as (CD45+CD64+CD11chigh Siglec-Fhigh), Mo-AM as (CD45+CD64+Siglec-FlowCD11c+CD11b+), and neutrophil as (CD45+CD11b+Ly-6G+).
a

## Slide 6
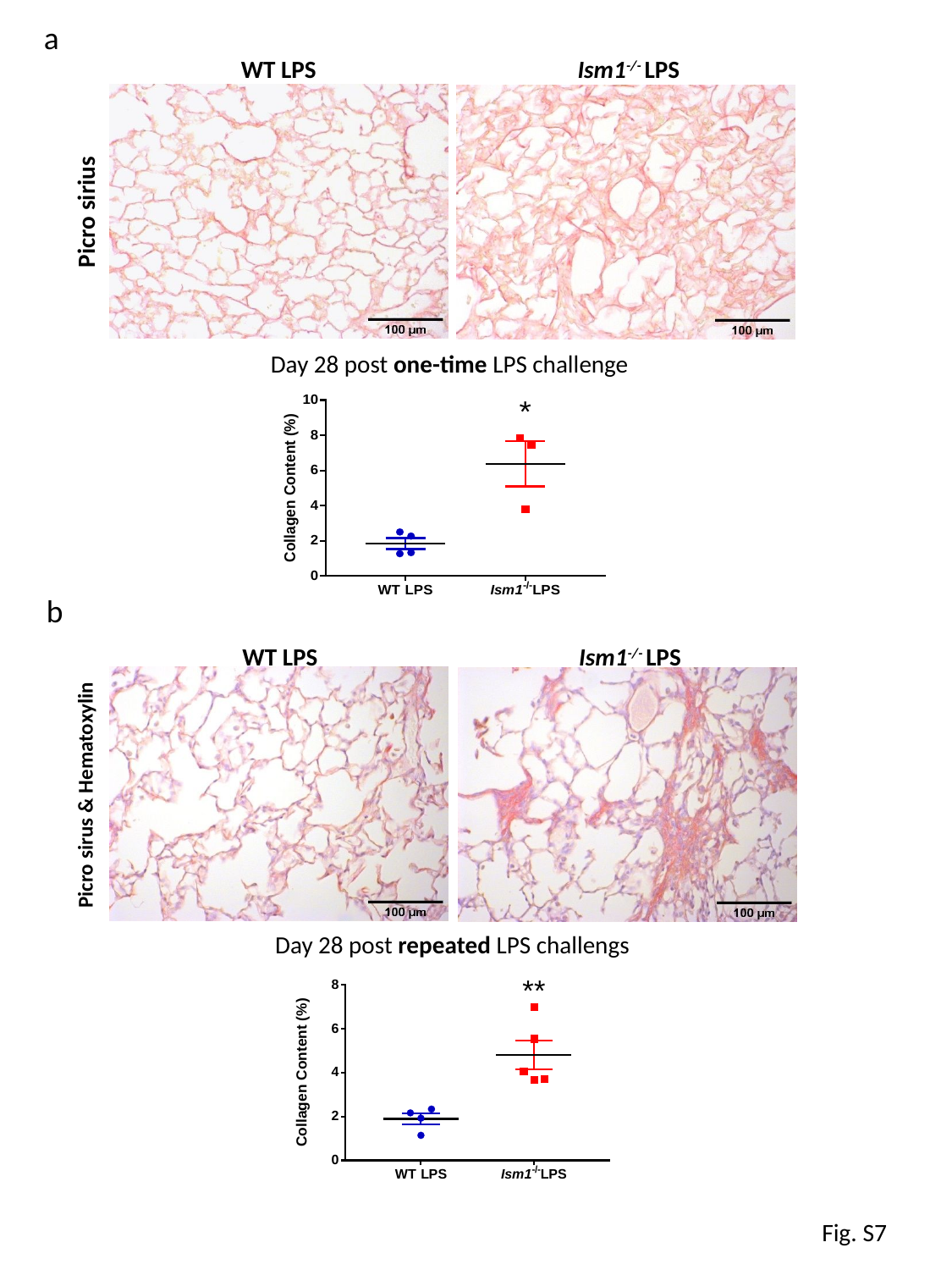

a
WT LPS
 Ism1-/- LPS
Picro sirius
Day 28 post one-time LPS challenge
b
WT LPS
 Ism1-/- LPS
Picro sirus & Hematoxylin
Day 28 post repeated LPS challengs
Fig. S7

## Slide 7
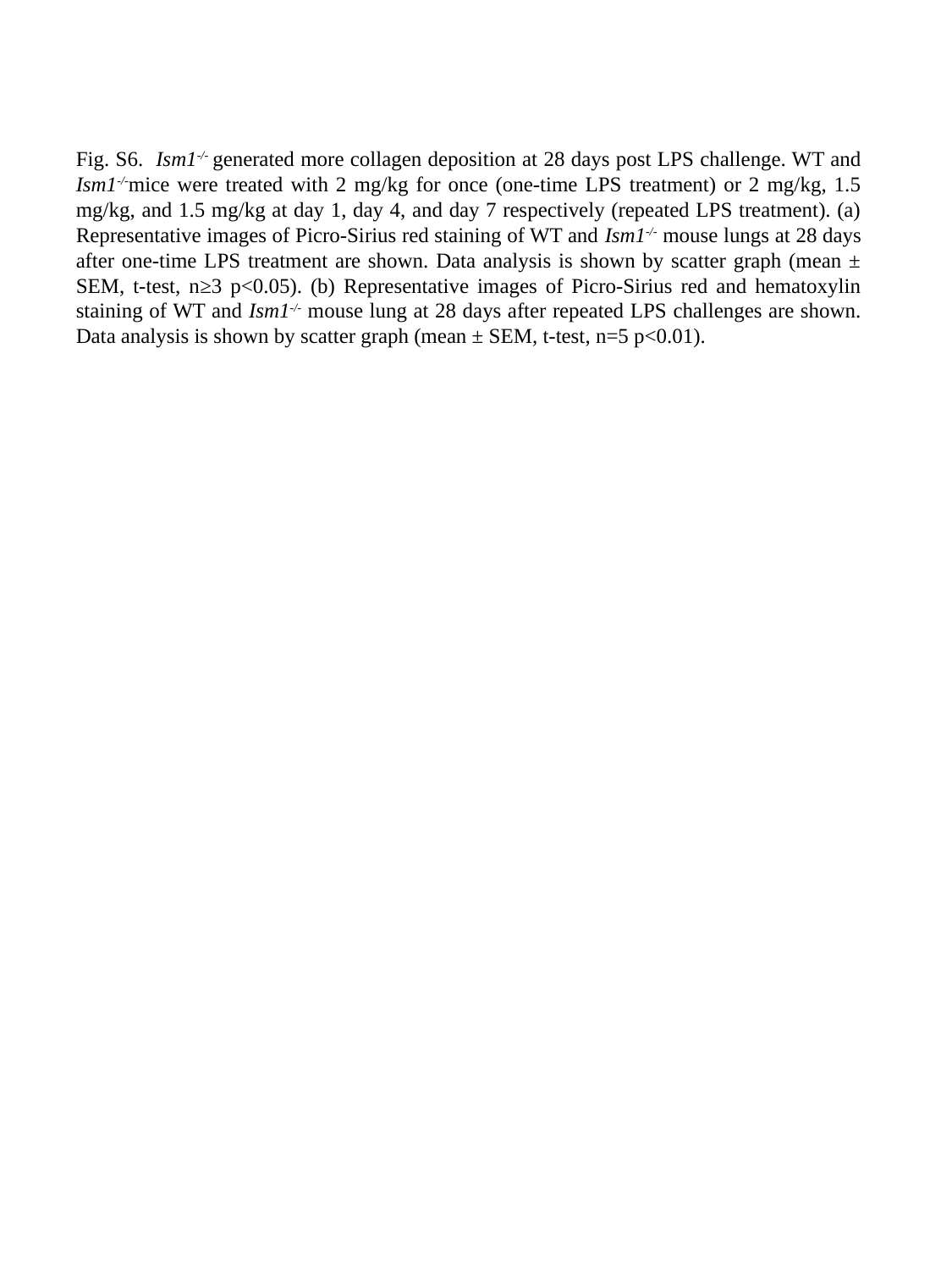

Fig. S6. Ism1-/- generated more collagen deposition at 28 days post LPS challenge. WT and Ism1-/-mice were treated with 2 mg/kg for once (one-time LPS treatment) or 2 mg/kg, 1.5 mg/kg, and 1.5 mg/kg at day 1, day 4, and day 7 respectively (repeated LPS treatment). (a) Representative images of Picro-Sirius red staining of WT and Ism1-/- mouse lungs at 28 days after one-time LPS treatment are shown. Data analysis is shown by scatter graph (mean ± SEM, t-test, n≥3 p<0.05). (b) Representative images of Picro-Sirius red and hematoxylin staining of WT and Ism1-/- mouse lung at 28 days after repeated LPS challenges are shown. Data analysis is shown by scatter graph (mean ± SEM, t-test, n=5 p<0.01).
